# The glucocorticoid RU24858 does not distinguish between transrepression and transactivation in primary human eosinophils

**DOI:** 10.1186/1476-9255-3-10

**Published:** 2006-07-12

**Authors:** Mirkka Janka-Junttila, Eeva Moilanen, Hannele Hasala, Xianzhi Zhang, Ian Adcock, Hannu Kankaanranta

**Affiliations:** 1 The Immunopharmacology Research Group, Medical School, FIN-33014 University of Tampere and Research Unit, Tampere University Hospital, Tampere, Finland; 2 Department of Thoracic Medicine, National Heart and Lung Institute, Imperial College, London, UK; 3 The Center for Infection and Immunity, Institute of Biophysics, Chinese Academy of Sciences, Beijing, China; 4 Department of Pulmonary Medicine Tampere University Hospital, Tampere, Finland

## Abstract

**Background:**

Glucocorticoids are used to treat chronic inflammatory diseases such as asthma. Induction of eosinophil apoptosis is considered to be one of the main mechanisms behind the anti-asthmatic effect of glucocorticoids. Glucocorticoid binding to its receptor (GR) can have a dual effect on gene transcription. Activated GR can activate transcription (transactivation), or by interacting with other transcription factors such as NF-κB suppress transcription (transrepression). RU24858 has been reported to transrepress but to have little or no transactivation capability in other cell types. The dissociated properties of RU24858 have not been previously studied in non-malignant human cells. As the eosinophils have a very short lifetime and many of the modern molecular biological methods cannot be used, a "dissociated steroid" would be a valuable tool to evaluate the mechanism of action of glucocorticoids in human eosinophils. The aim of this study was to elucidate the ability of RU24858 to activate and repress gene expression in human eosinophils in order to see whether it is a dissociated steroid in human eosinophils.

**Methods:**

Human peripheral blood eosinophils were isolated under sterile conditions and cultured in the presence and/or absence RU24858. For comparison, dexamethasone and mometasone were used. We measured chemokine receptor-4 (CXCR4) and Annexin 1 expression by flow cytometry and cytokine production by ELISA. Apoptosis was measured by DNA fragmentation and confirmed by morphological analysis.

**Results:**

RU24858 (1 μM) increased CXCR4 and Annexin 1 expression on eosinophils to a similar extent as mometasone (1 μM) and dexamethasone (1 μM). Like dexamethasone and mometasone, RU24858 did suppress IL-8 and MCP-1 production in eosinophils. RU24858 also increased spontaneous eosinophil apoptosis to a similar degree as dexamethasone and mometasone, but unlike dexamethasone and mometasone it did not reverse IL-5- or GM-CSF-induced eosinophil survival.

**Conclusion:**

Our results suggest that in human eosinophils RU24858 acts as transactivator and transrepressor like classical glucocorticoids. Thus, RU24858 seems not to be a "dissociated steroid" in primary human eosinophils in contrast to that reported in animal cells. In addition, functionally RU24858 seems to be a less potent glucocorticoid as it did not reverse IL-5- and GM-CSF-afforded eosinophil survival similarly to dexamethasone and mometasone.

## Background

Eosinophils are thought to play a critical role in allergic diseases, such as allergic rhinitis, asthma, and atopic dermatitis.[1] In patients with asthma, activation of eosinophils is thought to cause epithelial tissue injury, and increased bronchial responsiveness.[2] Apoptosis or programmed cell death is an important feature in the resolution of pulmonary inflammation.[3,4] Unlike necrosis which is characterized by loss of cell membrane integrity and the uncontrolled release of harmful cellular contents, apoptosis is characterized by formation of apoptic bodies, which are then phagocytosed intact so there will be no leakage of intracellular contents or activation of the inflammatory response.[3,5] Eosinophil apoptosis is inhibited by cytokines such as interleukin (IL)-3, IL-5 and granulocyte-macrophage colony-stimulating factor (GM-CSF) *in vitro *and *in vivo*.[1] In addition, we and others have previously shown that eosinophil apoptosis is delayed in patients with asthma or inhalant allergy.[6,7]

Glucocorticoids are potent anti-inflammatory agents for the treatment of allergic diseases such as asthma, allergic rhinitis, atopic dermatitis and various syndromes associated with hypereosinophilia. Enhancement of eosinophil apoptosis and/or reversal of cytokine-induced eosinophil survival have been reported to be one important mechanism by which glucocorticoids reduce eosinophil numbers. [8-18] The basic mechanism of glucocorticoid actions is that they penetrate into the cell and bind to glucocorticoid receptor molecules in the cytoplasm.[19,20] The glucocorticoid- glucocorticoid receptor (GR) complex acts as a transcription factor, binding to specific DNA sites in the nucleus. Within the nucleus, GR may induce gene transcription (transactivation) by binding to specific DNA sequences known as glucocorticoid response elements (GREs) in the promoter-enhancer regions of steroid responsive genes. The glucocorticoid-glucocorticoid receptor complex may also directly interact with other transcription factors such as nuclear factor-κB (NF-κB) and activator protein (AP)-1 resulting in a transcriptional down-regulation (transrepression), which is considered currently to be a major mechanism of the anti-inflammatory effect of steroids.[20,21] Despite many studies on the ability of glucocorticoids to repress and/or activate genes in other cell types [22-25] there are no published data on whether transactivation and/or transrepression events play a role in the regulation of apoptosis in human eosinophils.

Based on the hypothesis that the predominant anti-inflammatory effects of glucocorticoids derive from inhibition of transcription (transrepression), whereas the metabolic effects, like disrupting the regulation of calcium and glucose metabolism, derive from positive transcriptional effects (transactivation), experimental glucocorticoids, which only act as transrepressors but not as transactivators, have been developed. RU24858 is such a novel glucocorticoid.[22] However the transactivation profile of RU24858 has been controversial. Vayssiere et al.[22] and Vanden Berghe et al.[23] demonstrated RU24858 to be almost as effective as dexamethasone in inducing transrepression but show little or no transactivation ability in human and murine cell lines. In contrast, others have reported no dissociation between anti-inflammatory activity and side effects *in vivo*.[25] Differences between human and rodent GR or in GR-associated factors has been implicated to be critical for these divergent results.[24] However, the ability of RU24858 to dissociate between transactivation and transrepression in non-transformed primary human cells has not been described. Whether dissociated steroids such as RU24858 have a better safety profile in the treatment of chronic inflammatory diseases such as asthma depends on their ability to dissociate between transactivation and transrepression in non-malignant human cells.

Primary human eosinophils are terminally differentiated, non-dividing cells that can only be cultured for very short periods, making these cells unsuitable for many studies using molecular biology. Thus, a dissociative glucocorticoid would be a very valuable pharmacological tool to evaluate the mechanism of action glucocorticoids in primary cells such as human eosinophils. The aim of our study was to test whether RU24858 discriminates between transactivation and transrepression in human eosinophils and the functional consequences of this profile by assessing its effects on eosinophil apoptosis. We measured the induction of surface expression of Annexin I and CXCR4 as a measure of GR transactivation[26,27] ability and the inhibition of IL-8 and MCP-1 production to define the transrepression potential. We found that in eosinophils RU24858 possessed transrepression capability but also clear transactivation effects. Surprisingly, although RU24858 did result enhanced spontaneous eosinophil apoptosis it did not reverse cytokine-induced apoptosis like other glucocorticoids do.

## Methods

### Eosinophil isolation

Eosinophils were isolated under sterile conditions as previously reported.[6,17,18,28] Before donation of blood, all subjects gave informed consent to a study protocol approved by the ethical committee of Tampere University Hospital. Eosinophils were obtained from donors with eosinophil counts ranging from upper normal to slightly elevated. We excluded patients with hypereosinophilic syndrome. Venous blood (50–100 ml) was collected into 10–20 ml of acid citrate dextrose anticoagulant and hydroxyethyl starch solution. White blood cells were obtained after removing supernatant and were overlaid onto Ficoll and centrifuged at 700 g for 30 min at 20°C. Mononuclear cell layer was removed and the remaining pellet containing granulocytes and red blood cells was washed in HBSS (Hank's Balanced Salt Solution without Phenol Red). Red blood cells were lysed by hypotonic lysis.

Eosinophils were purified using immunomagnetic anti-CD16 antibody conjugated beads. Following separation, granulocytes were washed, counted and resuspended in 300 ml of RPMI 1640 (2% fetal calf serum and 5 mM EDTA). Cells mixed with beads were incubated at 4°C for at least 25 min before loading onto a separation column positioned within a magnetic field and washed with 40 ml of RPMI 1640. The eluted eosinophils were washed and counted using microscopic examination and diluting 10 μl cell suspension in 90 μl Kimura stain (consisting of 11 ml of 0.05% (wt/wt) toluidine blue, 0.8 ml of 0.03% light green SF yellowish, 0.5 ml of saturated saponin, and 5 ml of 0.07 M phosphate buffer, pH 6.4) and the purity of eosinophil population was >99%. The eosinophils were washed and resuspended at 1 × 10^6 ^cells/ml and cultured (37°C, 5 % CO_2_) in RPMI 1640 (Dutch modification, 10% fetal calf serum and antibiotics). Granulocytes were incubated in the presence and absence of RU24858, mometasone and dexamethasone. All the steroids were diluted in DMSO. The final concentration of DMSO in the cells was 0.1%. Similar concentration of DMSO was used in control experiments.

### Flow cytometry

Eosinophils were incubated for 24 h and the expression of CXCR4 was determined by using a PE-conjugated mAb against CXCR4 (20 μl/10^6 ^cells). We performed flow-cytometric analysis according to the instructions of the manufacturer and for comparison PE-conjugated isotype control was used. The expression of Annexin I was determined by using a mAb against Annexin I (20 μl/10^6 ^cells) and for comparison an isotype standard was used. As a secondary antibody we used PE-conjugated anti-mouse IgG_1 _monoclonal antibody according to that described by Liu et al.[29]

Unless otherwise stated the percentage of apoptotic cells was measured using a relative DNA fragmentation assay in propidium iodide stained cells by flow cytometry as previously described.[6,17,18,28] Eosinophils were incubated for 40 h. The cells showing decreased relative DNA content were considered as apoptotic.

### Morphological analysis

Cells were centrifuged onto cytospin slides. After fixation in methanol slides were stained with May-Grünwald-Giemsa. Cells showing typical features of apoptosis such as condensation of chromatin, nuclear coalescence and shrinkage of the cell were considered as apoptotic.[28,30] Cells were counted blind. We always prepared double identical samples and from each sample 200 cells were assessed and finally the average was calculated.

### Cytokine assays

Cytokine production was induced by 1 μM ionomycin.[31] Cells were incubated for 18 h and supernatants were collected and stored at -20°C and cytokines were measured by ELISA. The lower limits of detection were 3.9 pg/ml for MCP-1 and 4.1 pg/ml for IL-8.

### Materials

RU24858 was obtained from Aventis Pharma, Romainville Cedex, France and mometasone furoate was obtained from Schering-Plough, Kenilworth, USA. Dexamethasone and propidium iodide were purchased from Sigma Chemical Co. (St. Louis, MO). Other reagents were obtained as follows: antibiotics, fetal calf serum, RPMI 1640 (Gibco BRL, Paisley, Scotland, UK), anti-CD16 microbeads and magnetic cell separation system (Miltenyi Biotec Ltd., Surrey, UK), human recombinant IL-5, GM-CSF and DuoSet ELISA Development System for IL-8 and MCP-1 (R&D system Europe, Abingdon, UK), May-Grünwald (Merck, Darmstadt, Germany), and Giemsa (J.T. Baker, Deventer, Holland). PE-conjugated CXCR4 mAb (12G5), R-PE-conjugated IgG_2a _isotype control, Annexin I mAb, IgG_1 _isotype standard and R-PE-conjugated anti-mouse IgG_1 _monoclonal antibody were all purchased from BD Pharmingen (Temse, Belgium).

### Statistics

Data are expressed as mean ± SEM. Differences were analyzed by analysis of variance supported by Student-Newman-Keuls test and were considered significant if P < 0.05.

## Results

### The effect of RU24858 on CXCR4 expression on the surface of eosinophils

**Figure 1:**
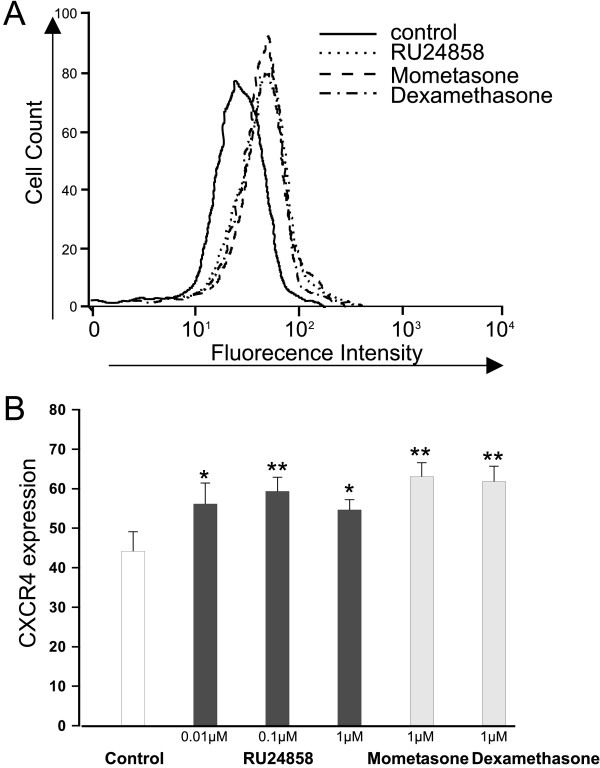
**The effects of RU24858, mometasone and dexamethasone on eosinophil CXCR4 surface expression**. Cells were stained and analyzed by using flow cytometry. A typical experiment showing an increase in CXCR4 expression following treatment of cells with mometasone, dexamethasone and RU24858 is indicated in (A). (B) Summary of results expressed as mean fluorescence intensity. Values are the mean ± S.E.M., n = 6. * Indicates P < 0.05, ** P < 0.01 as compared with the respective control in the absence of glucocorticoids.

Dexamethasone has previously been shown to induce CXCR4 expression in human eosinophils.[27] As expected, mometasone (1 μM) and dexamethasone (1 μM) induced CXCR4 expression in human eosinophils (Figure 1a &1b). To our surprise RU24858 (0.01–1 μM) also induced CXCR4 expression in a manner similar to classical glucocorticoids (Figure 1a &1b). To exclude the possibility that RU24858 affects the fluorescence properties of the PE-conjugated antibody, its effects were studied on cells labelled with PE-conjugated isotype control antibody. RU24858 (0.01 μM–1 μM), mometasone (1 μM) and dexamethasone (1 μM) had no effect on fluorescence of PE-conjugated isotype control (n = 6, data not shown).

### The effect of RU24858 on Annexin 1 expression on the surface of eosinophils

**Figure 2:**
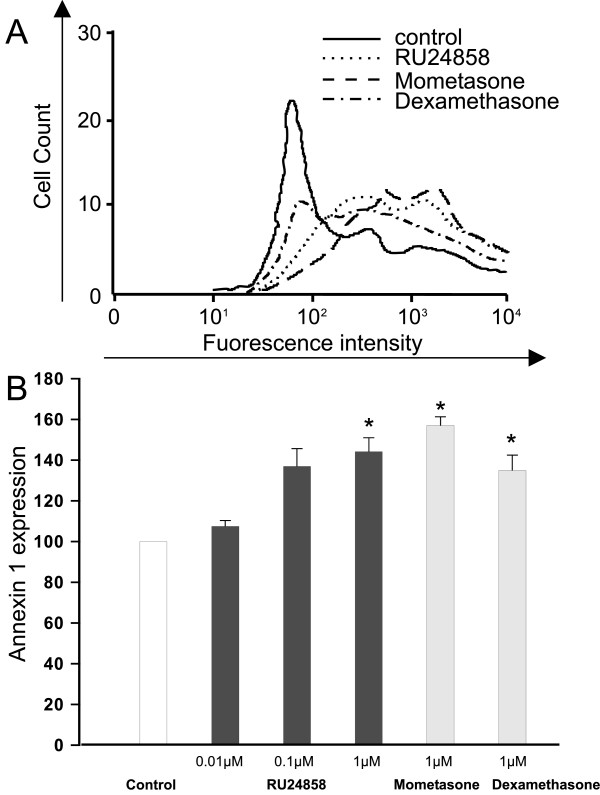
**The effects of RU24858, mometasone and dexamethasone on eosinophil Annexin 1 surface expression**. Cells were stained and analyzed by using flow cytometry. A typical experiment showing an increase in Annexin 1 expression following treatment of cells with mometasone, dexamethasone and RU24858 is indicated in (A). (B) Summary of results expressed as mean fluorescence intensity. Values are the mean ± S.E.M., n = 3. * Indicates P < 0.05 as compared with the respective control in the absence of glucocorticoids.

It has been recently demonstrated that glucocorticoids induce surface expression of Annexin 1 on human eosinophils.[29] To further analyse the transactivation ability of RU24858 we investigated whether it has a similar effect on Annexin 1 expression as mometasone and dexamethasone. RU24858 significantly increased Annexin 1 expression to a similar extent to that seen with mometasone and dexamethasone (both at 1 μM) (Figure 2a &2b). In addition, mometasone (1 μM), dexamethasone (1 μM) and RU24858 (0.01 μM–1 μM) had no effect on the fluorescence of isotype and/or secondary antibody controls (n = 6, data not shown).

### The Effect of RU24858 on cytokine production

**Figure 3:**
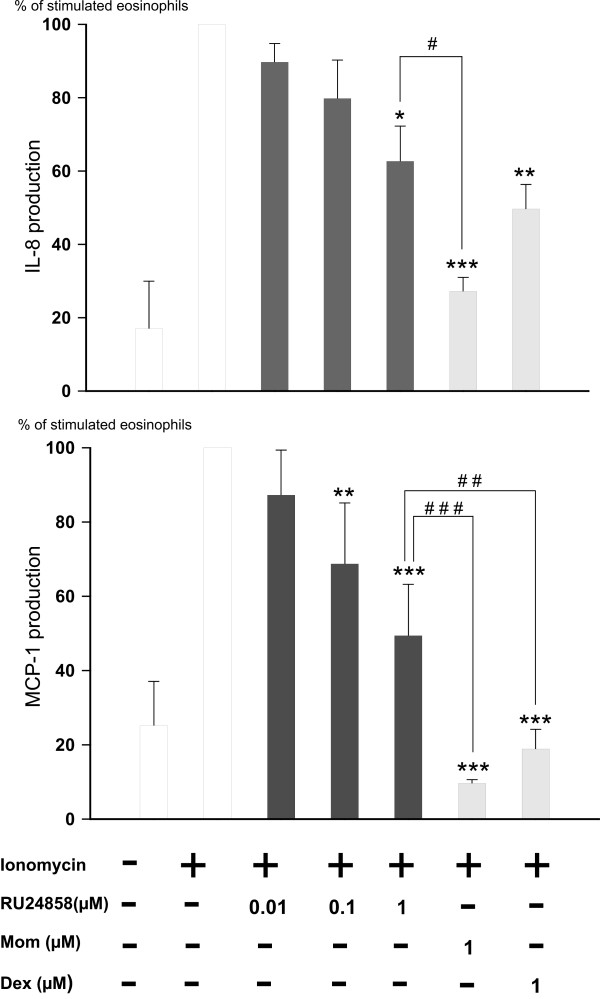
**The effects of RU24858, mometasone and dexamethasone on eosinophil cytokine production**. (A) IL-8 and (B) MCP-1. Ionomycin was used to stimulate the IL-8 and MCP-1 production in cells. IL-8 and MCP-1 were analyzed by ELISA based methods. Values are the mean ± S.E.M., n = 6. Results are expressed as % of stimulated eosinophils. * Indicates P < 0.05, ** P < 0.01 and *** P < 0.001 as compared with the respective control in the absence of glucocorticoids. # Indicates P < 0.05, ## P < 0.01 and ### P < 0.001 as compared with RU24858 (1 μM).

Glucocorticoids have previously been reported to inhibit IL-8 and MCP-1 production in eosinophils.[31] Therefore, to study the transrepression capability of RU24858 we measured IL-8 and MCP-1 production from supernatants collected from ionomycin-treated eosinophils. Spontaneous IL-8 and MCP-1 production was low and ionomycin (1 μM) increased MCP-1 and IL-8 production 6–10-fold (figure 3a &3b). Mometasone (1 μM) and dexamethasone (1 μM) inhibited IL-8 and MCP-1 generation as expected (figure 3a &3b) as also RU24858 (1 μM) did. The effect of RU24858 on MCP-1 production was significantly smaller than that of dexamethasone and mometasone. Mometasone was also more potent than RU24858 in inhibiting IL-8 production, whereas the difference between dexamethasone and RU24858 did not reach statistical significance (Figure 3a &3b). Taken together, the present data suggest that RU24858 is a non selective compound and does not dissociate between transactivation and transrepression in human eosinophils.

### Effects of RU24858 on cytokine-afforded eosinophil survival

**Figure 4:**
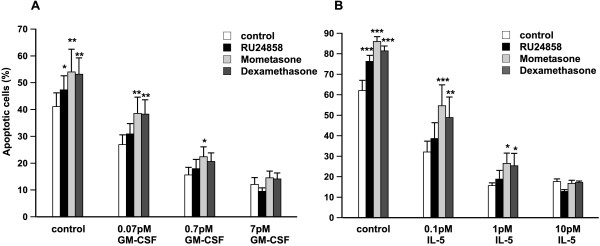
**The effects of RU24858, mometasone and dexamethasone on cytokine afforded eosinophil survival**. The effects of RU24858, mometasone and dexamethasone (all at 1 μM) on GM-CSF-(A) and IL-5-induced (B) eosinophil survival. Values are the mean ± S.E.M., n = 6. Results are expressed as percentage of apoptotic cells. * Indicates P < 0.05, ** P < 0.01 and *** P < 0.001 as compared with the respective control in the absence of glucocorticoids.

To evaluate the functional consequences of RU24858 actions, its effects on cytokine-induced eosinophil survival were measured. GM-CSF inhibited eosinophil apoptosis in a concentration-dependent manner (Figure 4a). GM-CSF (0.07 pM)-stimulated eosinophil survival was reversed by dexamethasone and mometasone (both at 1 μM) (Figure 4a). In contrast, RU24858 (1 μM) did not reverse GM-CSF-stimulated eosinophil survival (Figure 4a). When GM-CSF was added at increasing concentrations the effects of dexamethasone and mometasone reflected their GR agonist potency (Figure 4a). At 7 pM GM-CSF no glucocorticoid was able to attenuate eosinophil survival (Figure 4a).

IL-5 has also been reported to prolong eosinophil survival *in vitro*[32] and *in vivo*. IL-5 and GM-CSF receptors are composed of a common β-unit, but have unique α-units. This offers a possibility for differential signalling between IL-5 and GM-CSF.[1] IL-5 (0.1 – 10 pM) inhibited eosinophil apoptosis in a concentration-dependent manner, and its effects were partly reversed by dexamethasone and mometasone (figure 4b). This protection by glucocorticoids against IL-5-stimulated inhibition of apoptosis was lost at high concentrations of IL-5 (10 pM) (Figure 4b, Table 1). In contrast to dexamethasone and mometasone, RU24858 had no effect on IL-5-induced inhibition of eosinophil apoptosis (Figure 4b), which finding was further confirmed by using morphological analysis (Table 1). Taken together, in contrast to classical steroids, RU24858 does not reverse cytokine-afforded eosinophil survival.

**Table 1:** The effects of RU24858, mometasone and dexamethasone on IL-5 induced human eosinophil survival. Apoptosis was analyzed by morphological criteria. Results are expressed as percentage of apoptotic cells. Values are the mean ± S.E.M., n = 6. * Indicates P < 0.05, ** P < 0.01, *** P < 0.001 as compared with the respective control in the absence of glucocorticoids.

	**Apoptotic eosinophils (%)**		
	Control	1 nM IL-5	10 nM IL-5
control	43.3 ± 6.7	8.1 ± 1.5	9.3 ± 2.5
RU24858 1 μM	51.9 ± 5.5 *	11.6 ± 3.4	10.8 ± 3.8
Mometasone 1 μM	63.1 ± 6.8 ***	16.0 ± 2.9 **	10.8 ± 3.6
Dexamethasone 1 μM	60.1 ± 4.4 **	17.1 ± 3.3 **	7.7 ± 2.6

### Effects of RU24858 on spontaneous eosinophil apoptosis

**Figure 5:**
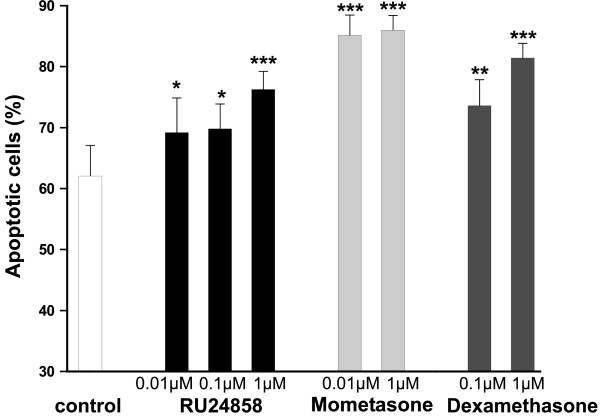
**The effects of RU24858, mometasone and dexamethasone on spontaneous eosinophil apoptosis**. The effects of RU24858 (0.01 μM, 0.1 μM, 1 μM), mometasone (0.01 μM, 1 μM) and dexamethasone (0.1 μM, 1 μM) on spontaneous eosinophil survival. Values are the mean ± S.E.M., n = 6. Results are expressed as percentage of apoptotic cells. * Indicates P < 0.05, ** P < 0.01 and *** P < 0.001 as compared with the respective control in the absence of glucocorticoids.

The currently used glucocorticoids enhance spontaneous eosinophil apoptosis at clinically relevant drug concentrations.[17,18] RU24858 increased spontaneous eosinophil apoptosis in a concentration-dependent manner similarly to that seen with dexamethasone and mometasone (Figure 5). Enhancement of spontaneous apoptosis by RU24858, dexamethasone and mometasone furoate was also confirmed by showing an increase in the number of eosinophils showing the typical morphological features of apoptotic eosinophils such as nuclear chromatin condensation and cell shrinkage (Table 1).

## Discussion

We report here for the first time the effects of a supposed "dissociated steroid" RU24858 in primary human cells. All the previous studies have been made with malignant human or murine cell lines. Our results show that the dissociated glucocorticoid RU24858 acts as a transrepressor in human eosinophils but also has transactivation capability. However, RU24858 seems to be somewhat less potent transrepressor than classical glucocorticoids dexamethasone and mometasone. Functionally, RU24858 is not able to reverse GM-CSF- or IL-5-induced eosinophil survival and thus has lower anti-inflammatory activity as compared with the classical glucocorticoids dexamethasone and mometasone.

It is currently proposed that the anti-inflammatory effects of glucocorticoids are due to inhibition of transcription, whereas many of the debilitating side-effects may derive from activation of transcription. So it has been hypothesised that it would be possible to separate the anti-inflammatory effects from the side-effects of glucocorticoids, by developing novel compounds which could repress inflammatory gene expression but not transactivate. RU24858 is such a novel glucocorticoid.

In contrast to our expectations, RU24858 induced transactivation in eosinophils as evidenced by increased CXCR4 and annexin 1 expression. This profile of activity is similar to that seen with classical glucocorticoids such as dexamethasone and mometasone. Interestingly, RU24858 increased CXCR4 expression at lower drug concentrations than Annexin I expression. To characterize the transrepression profile of RU24858 we measured cytokine production in eosinophils. As expected, dexamethasone, mometasone and RU24858 repressed IL-8 and MCP-1 production. However RU24858 was less potent in inhibiting cytokine production than the classical glucocorticoids. These results suggest that in human eosinophils RU24858 represses gene transcription as supposed, but also has an ability to transactivate. In our efforts to further characterize the ability of RU24858 to inhibit transcription, several cytokines and chemokines such as IL-6, RANTES and TNF-α reported to be suppressed by glucocorticoids in other cells[33,34] were measured. However, this was not successful as ionomycin was unable to induce the production of these cytokines in human eosinophils. Due to the inability to transfect primary human eosinophils we were unable to measure GRE- and κB-dependent reporter gene activity.

In the present study, we report that RU24858 was equipotent with classical glucocorticoids at inducing transactivation but was less potent at inducing transrepression. We have recently reported that RU24858 inhibits the expression of tristetraprolin at the level of mRNA and protein expression in J774 mouse macrophages at concentrations used in the present study.[35] In addition, we have shown that RU24858 inhibits LPS-induced inducible nitric oxide (iNOS) expression and nitric oxide production in mouse J774 macrophages and the effect of RU24858 was qualitatively and quantitatively similar to that of dexamethasone at similar drug concentrations.[36] Taken together, these results suggest that the ability of RU24858 to transactivate or transrepress depends on the cell type and source of cells. To the best of our knowledge, this is the first report on the effects of RU24858 in primary human cells. Studies in transfected cell lines can lead to anomalous conclusions. The receptors but also the signalling pathways that are present in a cell line can be different than in primary cells and can have a crucial effect on the results.[37]

In human and murine cell lines RU24858 has been reported to be almost as effective as dexamethasone in inducing transrepression but to show little or no transactivation ability.[22,23] In addition, only a weak transactivation in Hela cells was reported. In contrast, no difference in transactivation was seen between prednisolone and RU24858 in murine CV-1 cells.[24] In addition, no dissociation between the anti-inflammatory activity and side effects in *in vivo *animal models was reported. Thus, although it has previously been suggested that the profile of RU24858 is divergent and may be species and/or cell type specific, the effects of this compound have never been reported in primary human cells. A possible explanation for these divergent dissociation abilities of this compound is that RU24858 works as a partial agonist for glucocorticoid receptor. In cells where the numbers of receptors or signal transduction efficiency is high it works like a full agonist. In contrast, in cells where the number of receptors is low or efficiency of signal transduction is poor it may produce a maximal response that is less than that of the full agonist or it may even be an antagonist. Use of cells from different species can also lead to variability in results, as different species demonstrate divergent sensitivities to glucocorticoids. This may explain why Tanigava et al.[24] reported that RU24858 is able to transactivate in murine CV-1 cells but not in human HeLa cells. Thus, RU24858 might act as a full agonist glucocorticoid sensitive species, like mice, but in species less sensitive to glucocorticoids, like man, it would have a partial effect or even it might act as an antagonist. Another possible explanation for the conflicting results may be that one GR mRNA can produce up to at least eight functional GR isoforms.[38] These GR isoforms display diverse transcriptional activities and the levels of these isoforms vary among different cell types. Whether this cell type -specific isoform profile of GR activation can explain why RU24858 has divergent transcription profile depending on cell type remains to be determined.

Glucocorticoids are currently the most potent anti-inflammatory therapy available for the treatment of asthma.[19,20] One of the main anti-inflammatory effects of glucocorticoids in the treatment of asthma is the reduction in eosinophil numbers. One important mechanism by which glucocorticoids reduce eosinophil numbers seems to be the induction of eosinophil apoptosis and/or reversal of cytokine-afforded eosinophil survival.[11,13-15,17,18] Despite the fact that these mechanisms of glucocorticoids actions have been known for many years, the role of glucocorticoid-mediated transactivation or transrepression events in the regulation of eosinophil apoptosis has been unclear. Eosinophils are terminally differentiated, short-living and non-dividing cells that can be isolated only in low numbers. Thus, most of the modern molecular biology methods cannot be utilised to study signalling in human eosinophils. As glucocorticoids are known to up- or downregulate transcription of a number of genes in other cell types, a pharmacological tool with defined mechanism of action would be interesting in studying eosinophils. The main aim of our study was to find out if the "dissociated steroid" RU24858 could be a potent pharmacological tool in our efforts to identify whether the effects of glucocorticoids on eosinophil survival are due to activation of transcription or transrepression. To illustrate the problem, for example, of the 2409 genes studied in eosinophils, a total of 80 were found to be regulated 2-fold or greater after exposure to IL-5 for 1 h. Of these 73 were up-regulated and 7 were down-regulated.[39] Unfortunately, RU24858 does not dissociate between transactivation and transrepression in human eosinophils and thus is not suitable for evaluation of signalling in human eosinophils.

In the present study we report that RU24858 induces both transrepression and transactivation in human eosinophils. To analyze the functional consequence of this profile, we measured the effect of RU24858 on spontaneous apoptosis and cytokine-afforded survival of human eosinophils. Glucocorticoids are known to enhance spontaneous eosinophil apoptosis and to reverse cytokine-induced eosinophil survival.[17,18] RU24858 enhanced spontaneous eosinophil apoptosis, but unlike dexamethasone and mometasone it did not reverse IL-5- or GM-CSF-induced eosinophil survival. The result that the effect of dexamethasone and mometasone diminishes as the concentration of IL-5 or GM-CSF is increased is something that could be expected for compounds acting as "physiological antagonists". Rather than reflecting a true difference in the transactivation or transrepression profile between RU24858 and dexamethasone, the lack of effect of RU24858 on cytokine-induced eosinophil survival may be due to the partial agonistic properties and lower potency of RU24858 as compared with dexamethasone. However, as RU24858 does not reverse cytokine-induced survival of human eosinophils to a significant extent, it can be expected to be less potent in the treatment of asthma than the currently used glucocorticoids.

## Conclusion

In conclusion, we report here for the first time that RU24858 does not dissociate between transactivation and transrepression in non-malignant human cells, eosinophils.

## Abbreviations

AP-1: Activator protein 1

COPD: Chronic obstructive pulmonary disease

CXCR4: Chemokine receptor 4

GM-CSF: Granulocyte macrophage-colony stimulating factor

GR: Glucocorticoid receptor

GRE: Glucocorticoid response element

IL: Interleukin

MCP-1: Monocyte chemoattractant protein 1

NF-κB: Nuclear factor κB

## Competing interests

The author(s) declare that they have no competing interests.

## Authors' contributions

MJ carried out the eosinophil isolation, flow cytometric assays, morphological analyses, statistics and drafted the manuscript. EM participated in the design of the study and helped to draft the manuscript. HH gave valuable collaboration in laboratory and in apoptosis studies. XZ and IA gave assistance in the design and drafting of the manuscript. HK conceived the study, and participated in its design and coordination and helped to draft the manuscript. All authors read and approved the final manuscript.
